# Supplementary material for: Nano-Dimensional Carbon Nanosphere Supported Non-Precious Metal Oxide Composite: A Cathode Material for Sea Water Reduction
Source: Nanomaterials (Basel). 2022 Dec 6;12(23):4348. doi: 10.3390/nano12234348 (PMC9736246; doi:10.3390/nano12234348)
Supplement: Supplementary file 1 [file nanomaterials-12-04348-s001.zip › nanomaterials-2071249-supplementary.pdf]

Supplementary Materials

Nano-Dimensional Carbon Nanosphere Supported Non-Precious Metal Oxide Composite: A Cathode Material for Sea Water Reduction

Jayasmita Jana, Tran Van Phuc, Jin Suk Chung, Won Mook Choi * and Seung Hyun Hur *

School of Chemical Engineering, University of Ulsan, Daehak-ro 93, Nam-gu, Ulsan 44610, Republic of Korea

* Correspondence: wmchoi98@ulsan.ac.kr (W.M.C.); shhur@ulsan.ac.kr (S.H.H.); Tel.: +82-52-259-1065 (W.M.C.); +82-52-259-1028 (S.H.H.); Fax: +82-52-259-1689 (W.M.C. & S.H.H.)

1. Experimental Section

1.1. Materials

The commercial Pt/C, sodium chloride, and all chemicals for material synthesis were obtained from Sigma Aldrich. copper(II) chloride dihydrate ($CuCl_2.2H_2O$), cobalt(II) chloride hexahydrate ($CoCl_2.6H_2O$), urea, and triammonium citrate (TAC) were used for catalyst synthesis. The Platinum on graphitized carbon with at 20% loading (Pt/C) was used as the commercial catalyst for commercial reference. Ethanol, isopropanol, and potassium hydroxide were purchased from SK Chemicals, Korea. Buffer solutions were purchased from Daejung Chemicals. Nafion was from Sigma Aldrich. All chemicals were AR grade and were used as received. Distilled water was used throughout the experiment.

1.2. Characterization

The X-ray diffraction (XRD) pattern of all the samples were recorded in the 2θ range of 10–90° using a Rigaku X-ray diffractometer (D/MAZX 2500V/PC, Japan) with Cu Kα radiation (0.154 nm). The morphologies of the as-synthesized catalyst surfaces were observed through a field emission scanning electron microscope (JEOL JSM-6500 F, Japan) and a high-resolution transmission electron microscope (JEOL JEM-2100 F, an operating voltage = 200 kV). The surface analyses of the samples were performed on a Thermo Fischer Scientific ESCALAB 250 Xi (USA) using a monochromatic Al Kα as a radiation source (1486.6 eV). The nitrogen adsorption-desorption isotherms were recorded at 77 K using a Quantachrome Quadrasorb SI automated surface area and pore size analyzer to determine the surface area and the average pore diameters of the as-synthesized catalysts.

2. Results

2.1. Figures

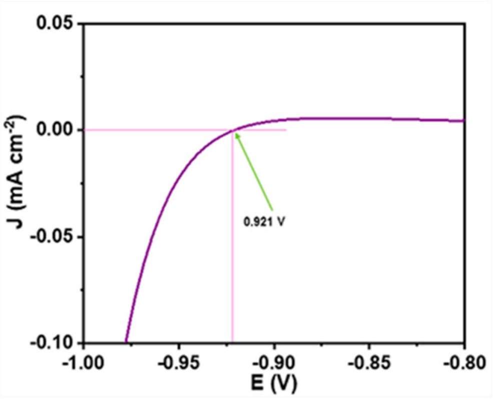

Figure S1. Calibration of Hg/Hg0 electrode in 1 M KOH solution (saturated with 5% H_2/Ar gas) at a scan rate of 2 mV/s.

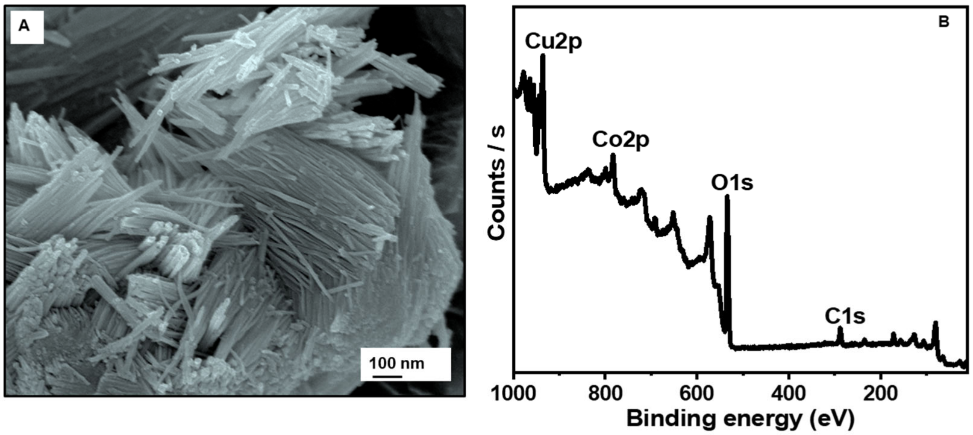

Figure S2. (A) SEM image of CCuU before calcination. (B) Broad range XPS spectra of CCuU. .

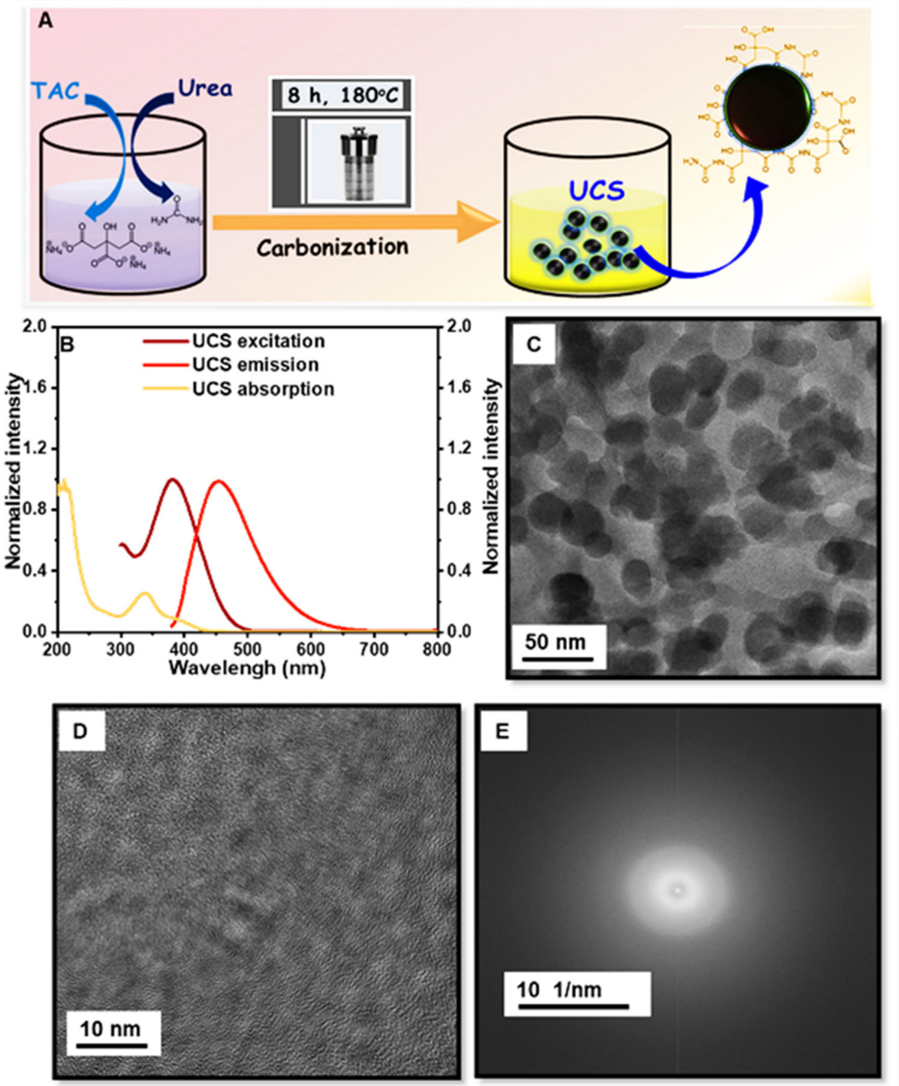

Figure S3. (A) Schematic of UCS synthesis. (B) Spectral profile, (C) TEM, (D) HRTEM, (E) FFT image of UCS particles.

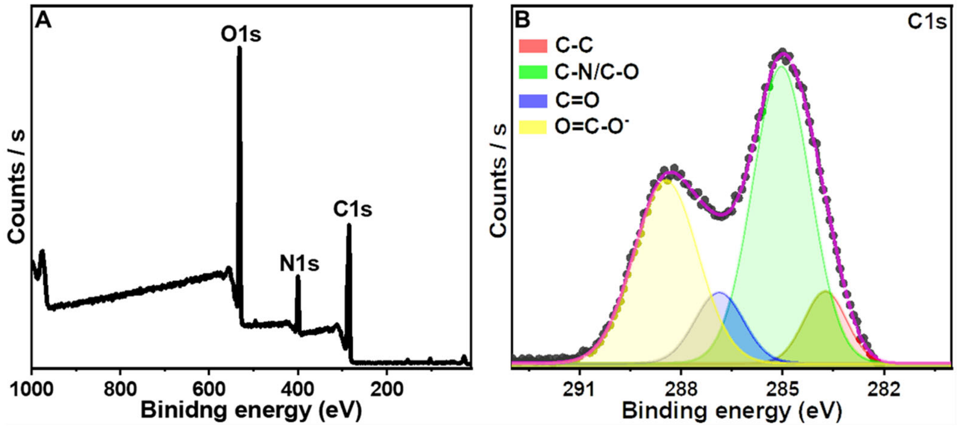

Figure S4. XPS (A) Broad range spectra and (B) Elemental analysis of C1s of UCS.

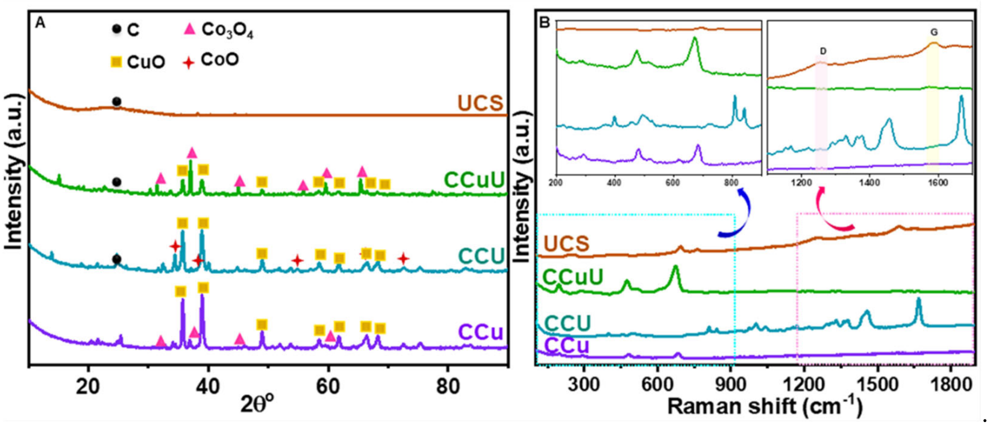

Figure S5. (A) XRD pattern and (B) Raman spectra of UCS, CCuU, CCU, and CCu.

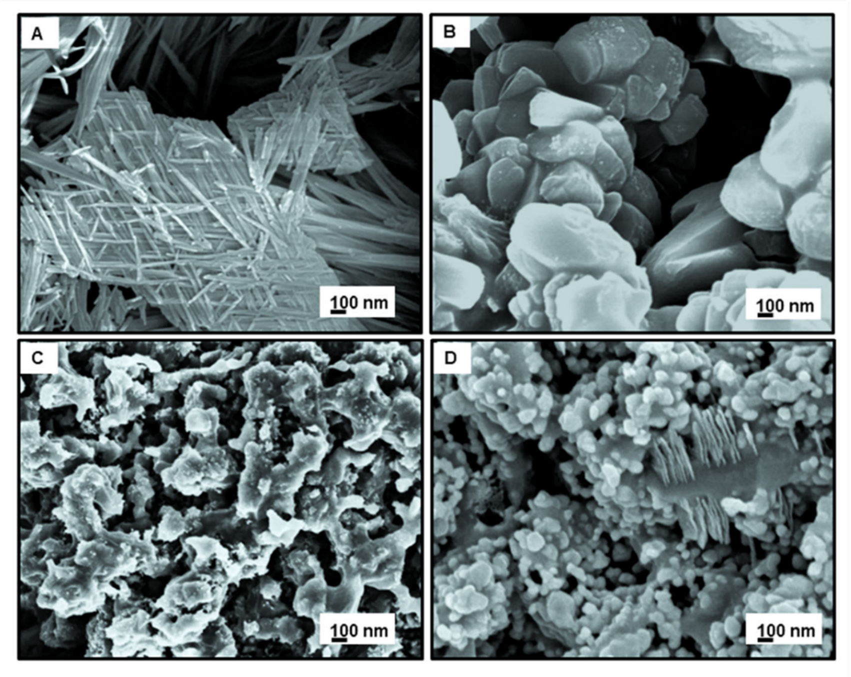

Figure S6. SEM images; before calcination (A) CCu and (B) CCU and after calcination (C) CCu, and (D) CCU.

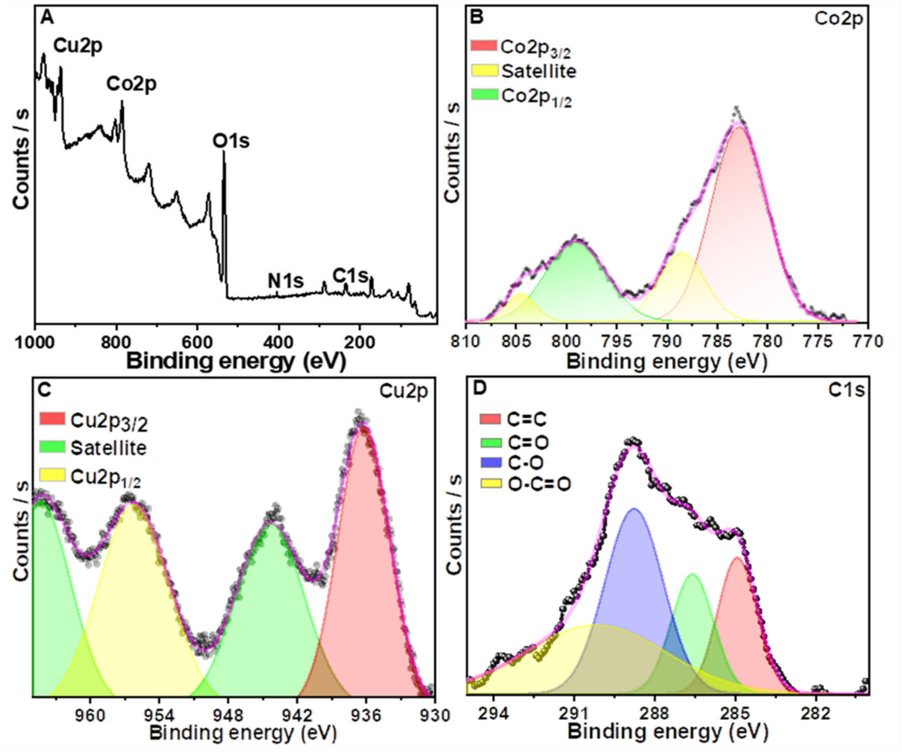

Figure S7. (A) Broad range spectra of CCu. Elemental analysis of (B) Co2p, (C) Cu2p, and (D) C1s.

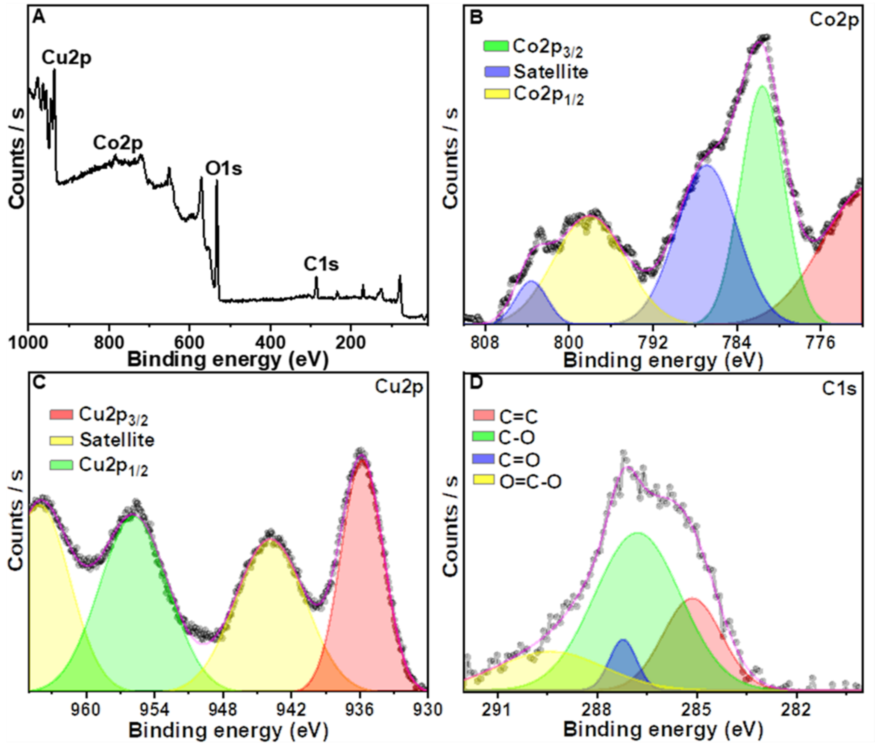

Figure S8. (A) Broad range spectra of CCU. Elemental analysis of (B) Co2p, (C) Cu2p, and (D) C1s.

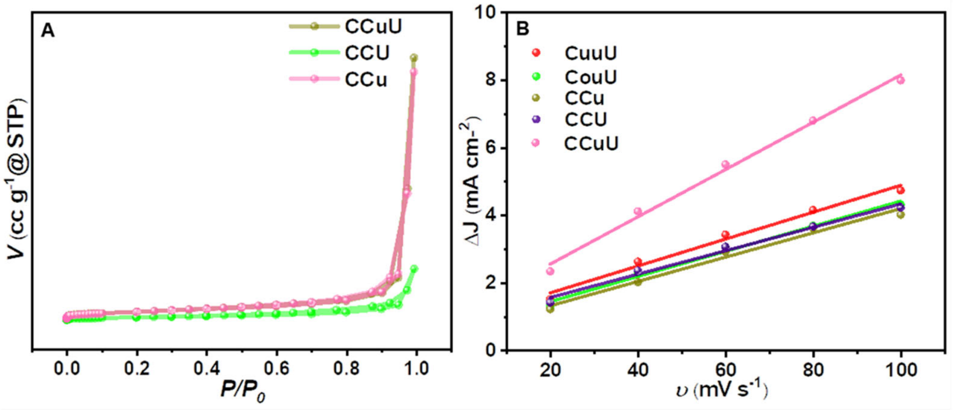

Figure S9. (A) BET isotherm of CCuU, CCU, and CCu. (B) Capacitive current density $\Delta J_{0.35\ V}$ as a function of scan rate (v) in the range of 0.1 to 0.4 V versus RHE for as-synthesized catalysts.

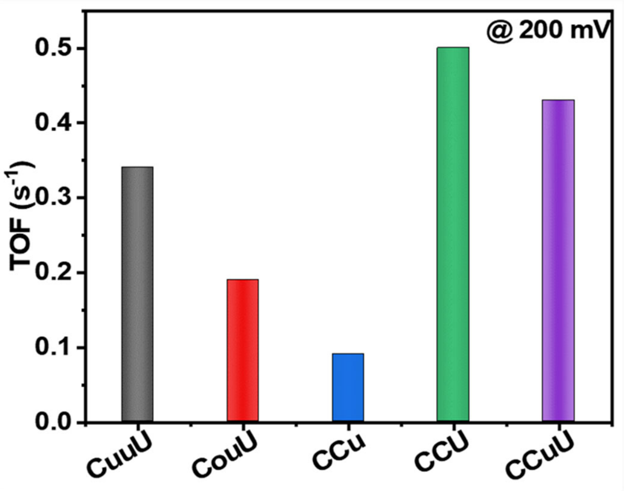

Figure S10. TOF values of materials in alkaline seawater during HER. The TOF values were calculated at 200 mV.

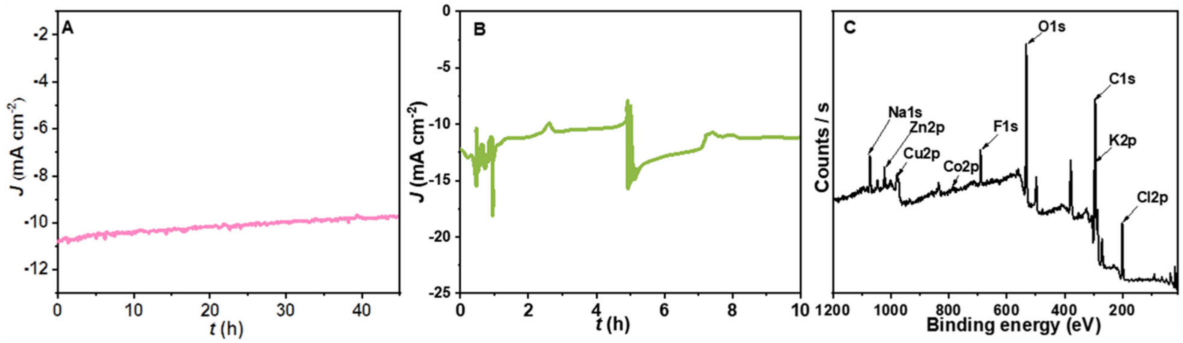

Figure S11. Chronoamperometric test of (A) CCuU over 40 h, and (B) Pt/C over 10 h in alkaline seawater at the overpotentials of 85 mV and 220 mV, respectively. (C) Broad range XPS spectrum of CCuU after 10 h scanning.

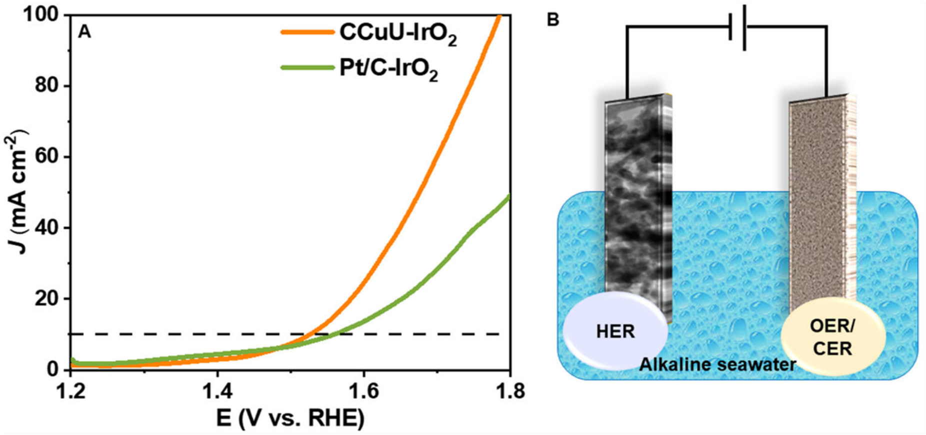

Figure S12. (A) Polarization curves, and (B) Schematic illustration of the two-electrode cell containing CCuU/Nifoam and Pt/C/Nifoam (individual) as the cathode and IrO_2/Nifoam as the anode for two-electrode system.

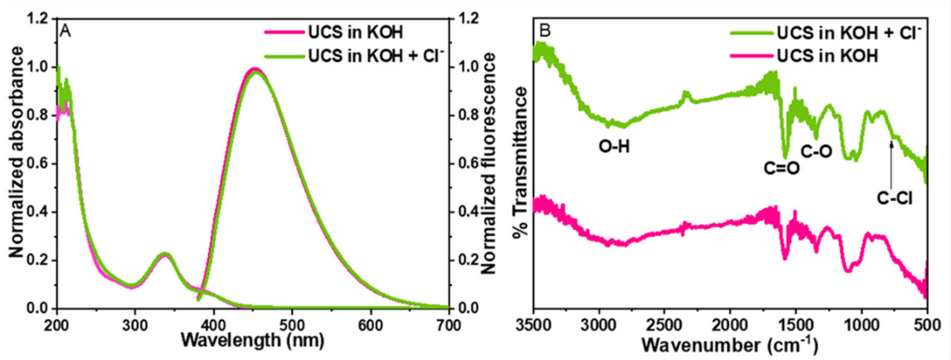

Figure S13. (A) Fluorescence spectral profile and (B) FT-IR spectra of alkaline UCS in absence and presence of chloride ions.

2.2. Tables

Table S1. Atomic % of the elements as obtained from the XPS.

Material	Atomic %								
	C1s	O1s	N1s	Cu2p	Co2p	Na1s	Cl2p	K2p	Zn2p
UCS	55.91	33.73	10.36	-	-	-	-	-	-
CCu	25.31	47.06	4.25	17.89	5.49	-	-	-	-
CCU	14.59	54.89	7.91	13.98	8.63	-	-	-	-
CCuU	17.72	52.72	3.43	19.20	7.40	-	-	-	-
CCuU post-cat	15.11	43.21	1.86	9.68	3.76	5.79	16.68	2.91	1.04

Table S2. Surface area analysis of composites.

Catalyst	ECSA / cm^2	BET surface area / m^2 g^{-1}	BET pore volume /cc g^{-1}
CCu	432.5	27.132	0.342
CCU	500	9.72	0.0706
CCuU	755	27.220	0.362

Table S3. The comparative account of HER activity by different electrocatalysts.

Catalyst	Electrolyte	Overpotential @ 10 mA cm^{-2}	Tafel slope (mV dec^{-1})	Ref.
Mn-NiO-Ni/Ni-F	Natural seawater	0.17 V	121	[1]
$PtNi_5$	Natural seawater	0.38 V@7.7 mA/g	119	[2]
NiCoN∣NixP∣NiCoN Electrode	Natural seawater	165 mV	139.2	[3]
Ni-SN@C	KOH + seawater	23 mV	39	[4]
Ti foil supported RuCo	Seawater	387	107	[5]
Fe–Co_2P BNRs	Seawater (pH = 7.8)	489	-	[6]
Fe-MoS2 nanosheet	Buffered seawater (pH = 7)	119	90	[7]
Mo_5N_6 Nanosheets	Ar-saturated natural seawater	94	66	[8]
**Carbon nanosphere supported CuO/Co_3O_4**	**Seawater in 1 M KOH**	**73 mV**	**47**	**Present Work**

References

1. Lu, X.; Pan, J.; Lovell, E.; Tan, T.H.; Ng, Y.H.; Amal, R. A Sea-Change: Manganese Doped Nickel/Nickel Oxide Electrocatalysts for Hydrogen Generation from Seawater. *Energy Environ. Sci.* **2018**, *11*, 1898–1910, doi:10.1039/c8ee00976g.
2. Zheng, J. Seawater Splitting for High-Efficiency Hydrogen Evolution by Alloyed PtNi x Electrocatalysts. *Appl. Surf. Sci.* **2017**, *413*, 360–365, doi:10.1016/j.apsusc.2017.03.285.
3. Yu, L.; Wu, L.; Song, S.; McElhenny, B.; Zhang, F.; Chen, S.; Ren, Z. Hydrogen Generation from Seawater Electrolysis over a Sandwich-like NiCoN∣NixP∣NiCoN Microsheet Array Catalyst. *ACS Energy Lett.* **2020**, *5*, 2681–2689, doi:10.1021/acsenergylett.0c01244.
4. Jin, H.; Wang, X.; Tang, C.; Vasileff, A.; Li, L.; Slattery, A.; Qiao, S.Z. Stable and Highly Efficient Hydrogen Evolution from Seawater Enabled by an Unsaturated Nickel Surface Nitride. *Adv. Mater.* **2021**, 2007508, doi:10.1002/adma.202007508.
5. Niu, X.; Tang, Q.; He, B.; Yang, P. Robust and Stable Ruthenium Alloy Electrocatalysts for Hydrogen Evolution by Seawater Splitting. *Electrochim. Acta* **2016**, *208*, 180–187, doi:10.1016/j.electacta.2016.04.184.
6. Lin, Y.; Sun, K.; Chen, X.; Chen, C.; Pan, Y.; Li, X.; Zhang, J. High-Precision Regulation Synthesis of Fe-Doped Co_2P Nanorod Bundles as Efficient Electrocatalysts for Hydrogen Evolution in All-PH Range and Seawater. *J. Energy Chem.* **2021**, *55*, 92–101, doi:10.1016/j.jechem.2020.06.073.
7. Huang, W.; Zhou, D.; Qi, G.; Liu, X. Fe-Doped MoS_2 Nanosheets Array for High-Current-Density Seawater Electrolysis. *Nanotechnology* **2021**, *32*, 415403, doi:10.1088/1361-6528/ac1195.
8. Jin, H.; Liu, X.; Vasileff, A.; Jiao, Y.; Zhao, Y.; Zheng, Y.; Qiao, S.Z. Single-Crystal Nitrogen-Rich Two-Dimensional Mo 5 N 6 Nanosheets for Efficient and Stable Seawater Splitting. *ACS Nano* **2018**, *12*, 12761–12769, doi:10.1021/acsnano.8b07841.
